# Supporting data for characterization of non-coding RNAs associated with the Neuronal growth regulator 1 (NEGR1) adhesion protein

**DOI:** 10.1016/j.dib.2016.02.053

**Published:** 2016-02-27

**Authors:** Prameet Kaur, Jun Rong Tan, Dwi Setyowati Karolina, Sugunavathi Sepramaniam, Arunmozhiarasi Armugam, Tsun-Hon Peter Wong, Kandiah Jeyaseelan

**Affiliations:** a Department of Biochemistry, Yong Loo Lin School of Medicine, National University of Singapore, 8 Medical Drive, 117597 Singapore; b Department of Pharmacology, Yong Loo Lin School of Medicine, National University of Singapore, 10 Medical Drive, 117597 Singapore; c Department of Anatomy and Developmental Biology, School of Biomedical Sciences, Faculty of Medicine, Nursing and Health Sciences, Monash University, Clayton, Victoria 3800, Australia

**Keywords:** Bioinformatics, Long non-coding RNA, MicroRNA, Negr1, Regulation

## Abstract

Long non-coding RNAs and microRNAs control gene expression to determine central nervous system development and function. Neuronal growth regulator 1 (NEGR1) is a cell adhesion molecule that plays an important role in neurite outgrowth during neuronal development and its precise expression is crucial for correct brain development. The data described here is related to the research article titled “A long non-coding RNA, BC048612 and a microRNA, miR-203 coordinate the gene expression of Neuronal growth regulator 1 (NEGR1) adhesion protein” [1]. This data article contains detailed bioinformatics analysis of genetic signatures at the *Negr1* gene locus retrieved from the UCSC genome browser. This approach could be adopted to identify putative regulatory non-coding RNAs in other tissues and diseases.

**Specifications Table**

**Table:** 

Subject area	*Biology*
More specific subject area	*Non-coding RNA mediated regulation of gene expression*
Type of data	*Table and figure*
How data was acquired	*In silico analysis: UCSC Genome browser and RegRNA*
	*qPCR: Applied Biosystems 7500 sequence detection system*
Data format	*Raw and analyzed data*
Experimental factors	*Primary neuronal cultures on Day 6 were transfected with either LNA™ GapmeR against the BC048612 lncRNA or mammalian expression vector containing BC048612.*
Experimental features	*Total cellular RNA was used to quantify expression levels.*
Data source location	*Singapore*
Data accessibility	*Within this article*

**Value of the data**

• This data provides a comprehensive *in silico* analysis of the genetic signatures and expression pattern of long non-coding RNAs associated with the *Negr1* gene.
• This data is useful in understanding the regulatory relationship between non-coding RNA gene expression and *Negr1* gene expression.
• The methodology provided can be used to postulate regulatory relationships between long non-coding RNAs and proximal genes.
• This approach could be adopted to identify putative regulatory non-coding RNAs in other tissues and diseases which could be evaluated with further functional studies.

## 1. Data

**Fig. 1:**
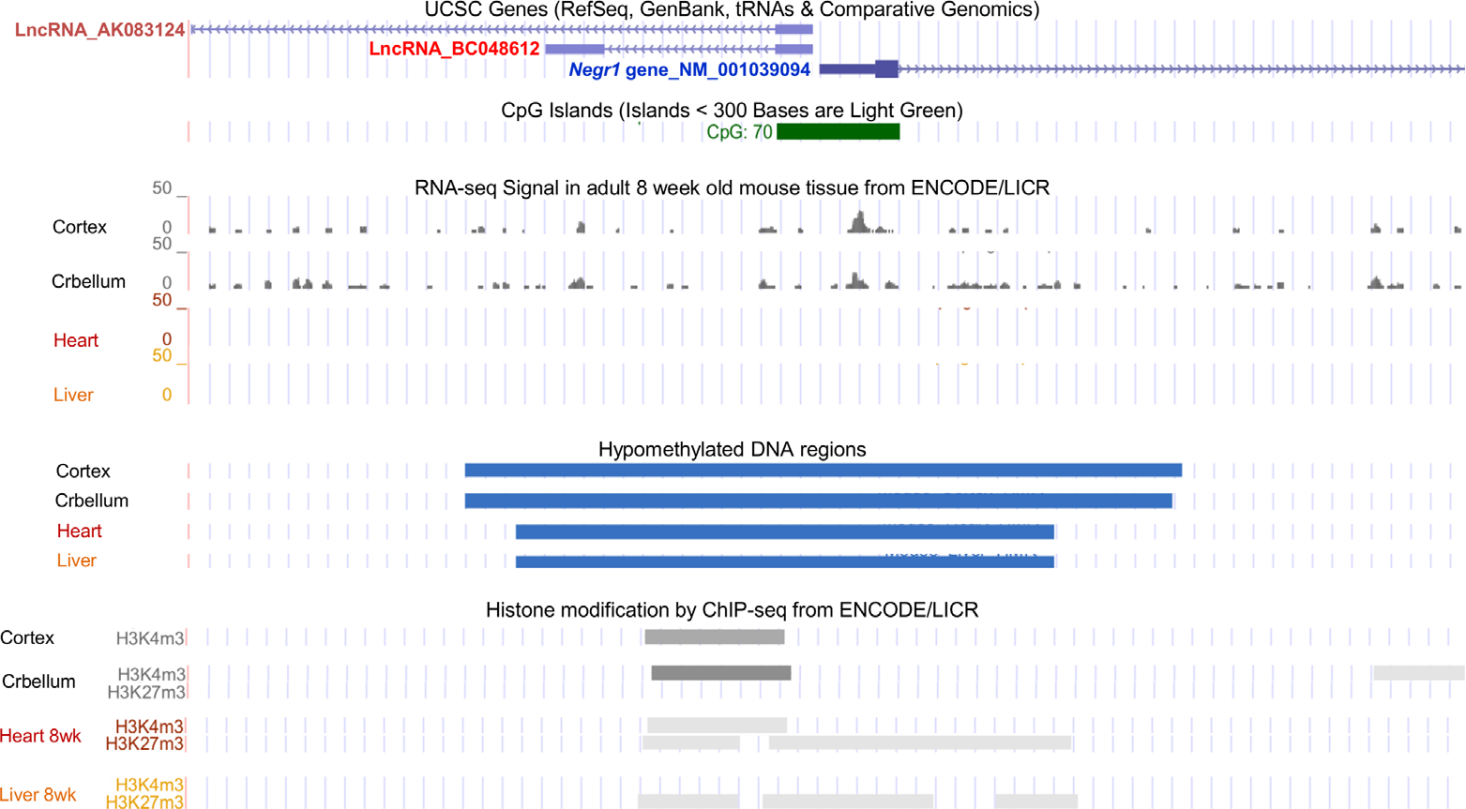
UCSC Genome Browser showing CpG islands, RNA sequencing data from ENCODE/LICR, DNA hypomethylation status and histone modification ChIP-seq data from ENCODE/LICR for the *Negr1* gene locus. Data in 8 week old mouse cortex, cerebellum, heart and liver was extracted from the UCSC Genome Browser [2], [3], [4], [5], [6], [7], [8], [9] on the Mouse July 2007 (NCBI37/mm9) assembly.

**Fig. 2:**
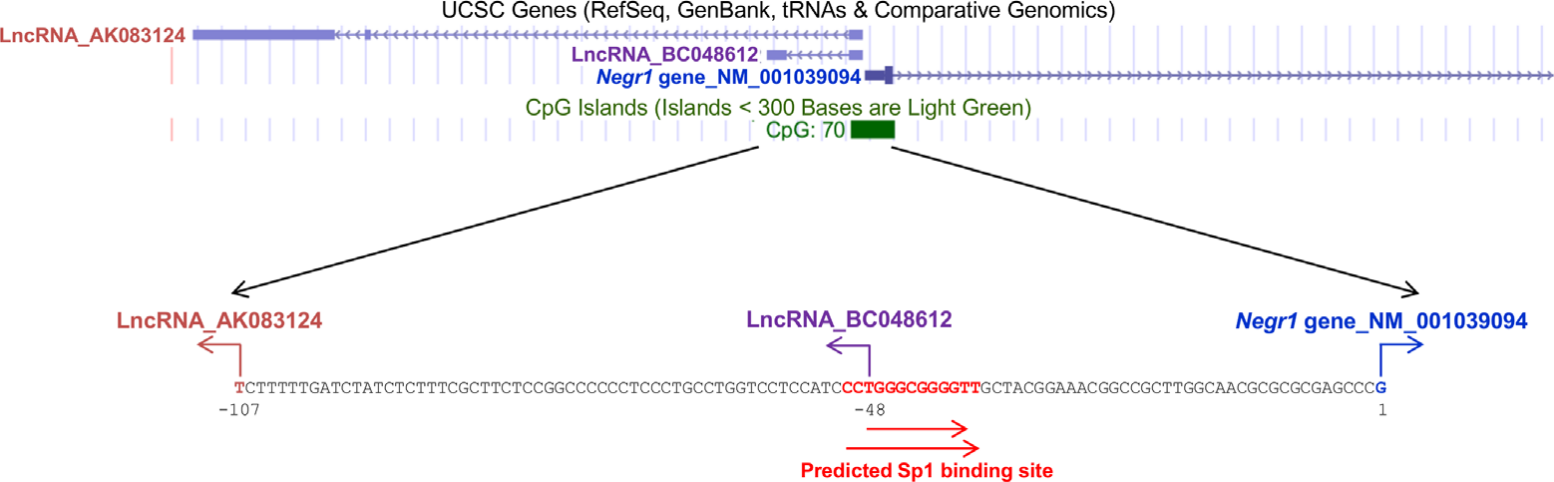
Mouse *Negr1* gene locus. UCSC genome browser showing *Negr1* gene and lncRNAs (AK083124 and BC048612) transcribed from the mouse *Negr1* bidirectional promoter based on the July 2009 mouse assembly. Refseq number for each transcript is indicated. CpG islands overlapping with the promoter are also indicated. Transcription start site of each transcript on the promoter sequence is denoted by letters in bold in specific colours with corresponding arrows denoting transcription direction. Putative Sp1 binding sites (red arrows) as determined by RegRNA [10] are mapped on the *Negr1* promoter.

**Fig. 3:**
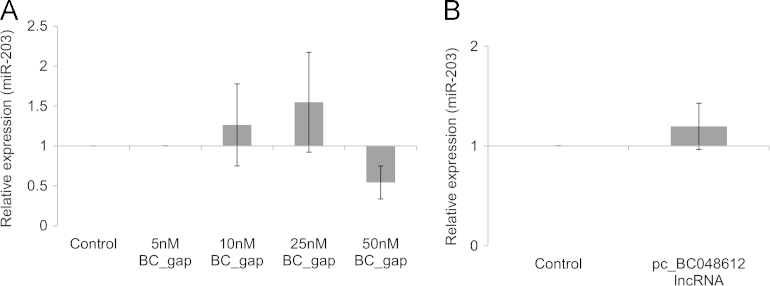
Effect of BC048612 lncRNA knockdown and over-expression on miR-203 expression. (A) Relative expression of miR-203 upon knockdown of BC048612 lncRNA in primary neuronal cultures. Expression was normalized to respective negative controls and GAPDH was used as a control/housekeeping gene to normalize miR-203 expression. Data are presented as mean±SD from 3 independent experiments. Statistical significance was tested using the Student׳s *t*-test. (B) Relative expression of miR-203 in murine primary cortical neuronal cultures over-expressing BC048612 lncRNA. Expression was normalized to cells transfected with empty vector. Data are presented as mean±SD from 3 independent experiments. Statistical significance was tested using the Student׳s *t*-test.

The data in this article describes the initial *in silico* analysis carried out on the *Negr1* gene locus for genetic signatures and expression patterns of long non-coding RNAs in proximity to the *Negr1* gene [1] (Fig. 1, Fig. 2, Table 1). The proposed interaction between the BC048612 lncRNA and microRNA-203 were validated using qPCR (Fig. 3).

## 2. Experimental design, materials and methods

### 2.1. Bioinformatics analysis of *Negr1* gene promoter

The *Negr1* gene promoter region was analyzed using the UCSC Genome Browser [2], [3]. Two lncRNAs, AK083124 and BC048612, were expressed from the *Negr1* gene promoter (Fig. 1). Presence of CpG islands [2], [3], expression profiles of the lncRNAs and *Negr1* mRNA in four different mouse tissues (8 week old cerebellum, cortex, heart and liver) [4], [5], [6], [7], DNA hypomethylation status [8], [9] as well as histone modification profiles by ChIP-seq from ENCODE/LICR were retrieved from the UCSC genome browser (Fig. 1). The findings from these analyses are summarized in Table 1.

Further interrogation of the BC048612 lncRNA and *Negr1* gene transcription start sites as well as putative regulatory transcription factors was carried out (Fig. 2). RegRNA [10] was employed to predict transcription factor binding sites proximal to the transcription start sites (Fig. 2).

### 2.2. Determination of effect of lncRNA expression on miR-203 levels

To determine if there is any interaction between the BC048612 lncRNA and miR-203, the BC048612 sequence was analyzed for putative binding sites for miR-203. RegRNA analysis did not predict any miR-203 binding sites on the BC048612 lncRNA [10]. Further verification of the effect of BC048612 lncRNA expression on miR-203 levels was carried out by quantifying miR-203 expression in both lncRNA knockdown (Fig. 3A) and over expression of lncRNA (Fig. 3B) in primary neuronal cultures. Primary neuronal cultures on Day 6 were transfected with LNA™ GapmeR against the BC048612 lncRNA or mammalian expression vector (pcDNA4/TO/myc His A) containing BC048612 (pc_BC048612 LncRNA). The cells were harvested 48 h after transfection and total cellular RNA was extracted for quantification of miR-203 expression levels.

## Competing interests

The authors declare that they have no competing interests.

## Figures and Tables

**Table 1:** *Negr1* mRNA, lncRNAs (AK083124 and BC048612) expression, DNA hypomethylation status, H3K4m3 and H3K27m3 histone modification data in 8 week old mouse cortex, cerebellum, heart and liver extracted from the UCSC Genome Browser [2], [3], [4], [5], [6], [7], [8], [9] on the Mouse July 2007 (NCBI37/mm9) assembly. Detailed information is presented in Fig. 1. N.A.: data not available.

	***Negr1*****mRNA expression**	**LncRNAs expression**	**DNA hypomethylation**	**H3K4m3**	**H3K27m3**
**Cortex**	+	+	++	+	N.A.
**Cerebellum**	+	+	++	+	−
**Heart**	−	−	+	+	+
**Liver**	−	−	+	−	+
