# Supplementary material for: Supporting data for characterization of non-coding RNAs associated with the Neuronal growth regulator 1 (NEGR1) adhesion protein
Source: Data Brief. 2016 Feb 27;7:381–5. doi: 10.1016/j.dib.2016.02.053 (PMC4781925; doi:10.1016/j.dib.2016.02.053)
Supplement: Supplementary file 1 — Supplementary material [file mmc1.doc]

**AUTHOR DECLARATION**

All the authors listed in this DIB declare that there are no conflict of interest associated with this publication.

Dr Prameet KAUR

Dr Jun Rong TAN

Dr DwiSetyowati KAROLINA

Dr Sugunavathi SEPRAMANIAM

Dr Arunmozhiarasi ARMUGAM

Professor Peter HT WONG

Professor Kandiah JEYASEELAN

Conflict of Interest-NONE
